# Concomitant use of antibiotics and immune checkpoint inhibitors in patients with solid neoplasms: retrospective data from real-world settings

**DOI:** 10.3332/ecancer.2020.1038

**Published:** 2020-05-08

**Authors:** Akhil Kapoor, Vanita Noronha, Vijay M Patil, Amit Joshi, Nandini Menon, Abhishek Mahajan, Amit Janu, Kumar Prabhash

**Affiliations:** 1 Department of Medical Oncology, Tata Memorial Hospital, Homi Bhabha National Institute, Mumbai, 400012, India; 2 Department of Radiodiagnosis, Tata Memorial Hospital, Homi Bhabha National Institute, Mumbai, 400012, India; a http://orcid.org/0000-0001-6006-2631

**Keywords:** immune checkpoint inhibitor, antibiotics, survival, real-world data

## Abstract

**Background:**

The use of antibiotics is known to alter the gut microbiome and it is hypothesised that the use of antibiotics may also alter the response to immune checkpoint inhibitors (ICI). As data is limited from real-world settings, we performed a retrospective audit of patients who received ICI along with concomitant antibiotics.

**Patients and Methods:**

This study is a retrospective audit of a prospectively collected the database of patients who received ICI for advanced solid tumours in any line between August 2015 and November 2018 at Tata Memorial Hospital, Mumbai, India. Antibiotic use was recorded from 2 weeks before the start of ICI and concomitantly with ICI. All statistical calculations were performed using Statistical Package for the Social Sciences (SPSS) statistical software for windows version 20.0.

**Results:**

A total of 155 patients were identified as having received ICI during the study period, out of which 70 (44%) patients received antibiotics. Median PFS in patients who received antibiotics was 1.7 months (95% CI: 1.1–2.3) as against 3.6 months (95% CI: 2.3–4.8) for patients who did not receive antibiotics (*p* = 0.912). Median OS in the patients who received antibiotics was 3.9 months (95% CI: 1.8–11.4) as compared to 9.2 months (95% CI: 4.2–12.3) who did not receive antibiotics *p* = 0.053 (HR = 1.023; 95% CI: 1.00–1.04). Among the patients who received antibiotics, median OS for patients who received ≤10 days of antibiotics was 8.8 months (95% CI: 4.2–11.2) while for patients receiving >10 days of antibiotics, it was 2.8 months (95% CI: 1.2–4.4), *p* = 0.025 (HR = 2.0, 95% CI: 1.1–3.7). Thirty-three (21.2% of total) patients received antibiotics during the window of 2 weeks before the start of ICI to 2 months of starting ICI. Median OS in the patients who received antibiotics in this window was 2.8 months (95% CI: 1.2–4.5) as compared to 9.2 months (95% CI: 5.2–13.1) who did not receive antibiotics *p* = 0.008 (HR = 1.8; 95%CI: 1.2–3.0).

**Conclusions:**

This study shows that the judicious use of antibiotics is required in patients on ICI or scheduled to be started on ICI.

## Introduction

The human gut microbiota represents a complex and interconnected ecosystem composed of trillions of microorganisms living within the human gut [1]. Preclinical data using mice with similar sex, age and genetic background have suggested that the immune anticancer activity of checkpoint inhibitors (ICI) was lost in the absence of immunogenic gut bacteria [2]. There have been reports that faecal microbiota transplantation (FMT) from cancer patients who responded to ICIs into antibiotic-treated mice restored the efficacy of PD-1 therapy while FMT from non-responder patients into antibiotic-treated mice failed to stimulate the PD-1 response [2]. There is time-dependent partial repopulation of the gut microbiota after antibiotic discontinuation which is important to be taken into account. In a study by Derosa *et al* [3], a negative association of antibiotics was observed on the clinical activity of ICI in patients with advanced renal and non-small-cell lung cancer both in terms of progression free survival (PFS) and overall survival (OS) [3]. Another study by Routy *et al* [2] studied a large cohort of patients with non-small cell lung cancer (NSCLC), renal and urothelial carcinomas and demonstrated that cases receiving antibiotics between 2 months before and 1 month after the first ICI administration had worse PFS and OS than their non-antibiotic treated counterparts. Furthermore, molecular characterisation of microbiota through shotgun sequencing of stool DNA led to conclusion that clinical response to ICI is correlated to the abundance of *Akkermansia muciniphila*. Gopalkrishnan *et al* [4] prospectively studied patients with metastatic melanoma treated with ICI and classified patients as responders if they achieved at least disease stability for 6 months. They found significant differences in the composition of bacterial flora between responders and non-responders. These studies point towards the role of microbiome in response to ICI therapy. The use of antibiotics is known to alter the gut microbiome and it is hypothesised that the use of antibiotics may also alter the response to ICI. We performed a retrospective audit of prospectively collected database of patients who received ICI along with concomitant antibiotics.

## Patients and methods

### Study population

This study is a retrospective audit of a prospectively collected the database of patients who received ICI for advanced solid tumours in any line between August 2015 and November 2018 at Tata Memorial Hospital, Mumbai, India. Antibiotic use (both oral and/or intravenous) for at least 5 days was recorded from 2 weeks before the start of ICI and concomitantly with ICI. Also, an additional analysis was performed to evaluate the use of antibiotics during the window of 2 weeks before to first 2 months versus other patients receiving ICI. The choice of antibiotics was based on the clinical and radiological focus for infection at presentation and subsequently modified based on response and culture reports. Steroid use was considered significant if patients received prednisolone equivalent of ≥10 mg per day for any duration. This particular threshold was in accordance with the exclusion criterion of most of the pivotal immunotherapy clinical trials [5, 6]. All these data were extracted from electronic medical records. The study was approved by the institutional ethics committee and review board.

### Clinical outcomes

The response assessment was performed using radiological assessment according to the Response Evaluation Criteria in Solid Tumours version 1.1. Response assessment was done 2 months after the commencement of ICI or at any symptoms/signs of clinical progression whichever was earlier. Adverse events during immunotherapy were documented and graded using the Common Terminology Criteria for Adverse Events, version 4.02. PFS was defined as the interval from the date of starting ICI till the date of progression or death due to any cause if it occurred before disease progression or the last follow-up date whichever was earlier. OS was calculated from the date of the start of ICI to date of death. Patients who were still alive were censored at the date of the last contact.

### Statistical analysis

Descriptive statistics were used to summarise categorical and continuous variables. Time-to-event analysis was done using the Kaplan–Meier estimator and hazard ratio was calculated by using the Cox proportional model. A two-way analysis of variance was conducted that examined the effect of use of steroids and use of antibiotics on the overall survival. All *p* values were based on a two-sided hypothesis with confidence interval (CI) at the 95% level, and *p* < 0.05 was considered as statistically significant. All statistical calculations were performed using SPSS statistical software for windows version 20.0 (Armonk, New York, IBM Corp.).

## Results

**Figure 1.**
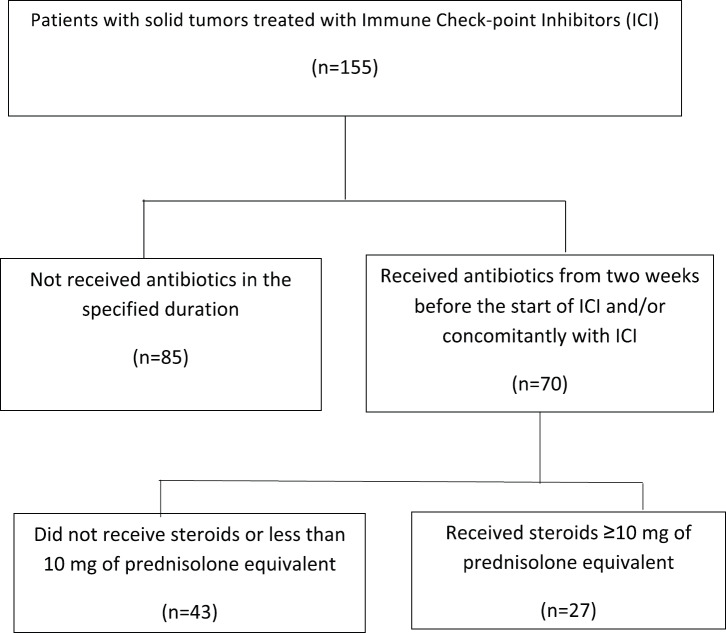
Consort diagram of the study.

A total of 155 patients were identified to have received ICI during the study period out of which 70 (44%) patients received antibiotics. All these patients had received nivolumab as ICI. Figure 1 shows the consort diagram of the study. The median age of the patients receiving antibiotics was 56.9 (range 30–83) years and 58 (82.9%) patients were males. ICI was used as first or second-line therapy in 36 (51.4%) patients and 6 (8.6%) patients had brain metastasis at the time of initiating ICI. Performance status as per ECOG was 0–1 in 37 (52.8%) patients, while steroids were required in 27 (38.6%) patients. The primary site was lung in 34 (63.1%) patients followed by head and neck in 20 (15.8%) patients. Other two most common histologies were urothelial cancer (10%) and conventional renal cell carcinoma (RCC, 8.6%). The median duration of antibiotic use was 10 (5–40) days. The duration of antibiotics use was ≤10 days in 41 (58.6%) and >10 days in 29 (41.4%) patients. Most common antibiotics used were beta-lactams in 49 (70%) patients, aminoglycosides in 13 (18.5%), fluoroquinolones in 23 (32.8%) and carbapenem in 6 (8.6%) patients.

**Figure 2.**
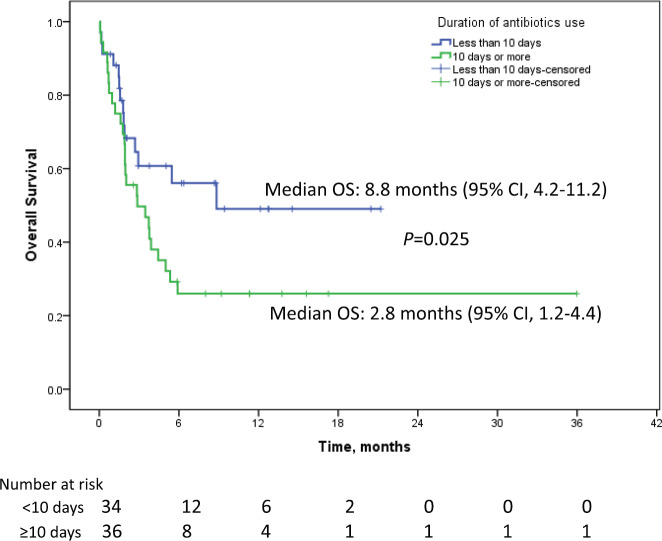
Kaplan–Meier curve showing overall survival in patients on immune checkpoint inhibitors who received antibiotics for ≤10 days (blue) versus >10 days (green).

**Figure 3.**
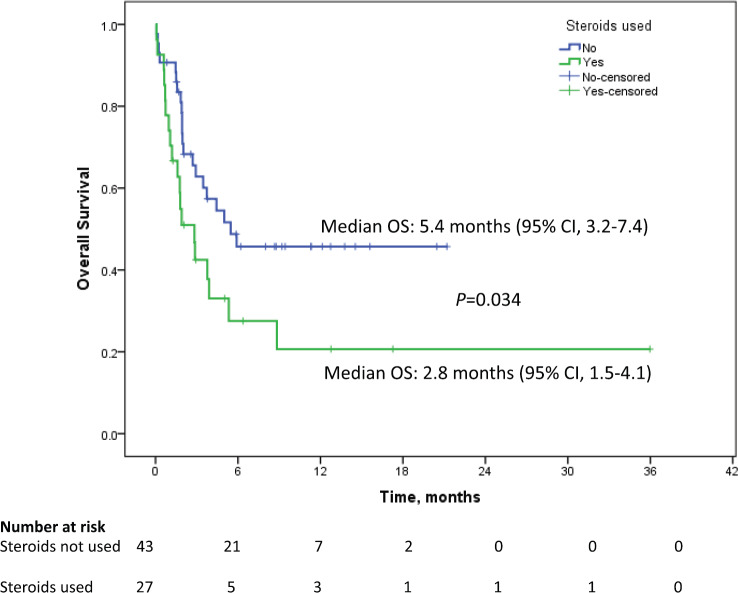
Kaplan–Meier curve showing overall survival in patients on immune checkpoint inhibitors who received concomitant steroids along with antibiotics (green) versus who received only antibiotics (blue).

**Figure 4.**
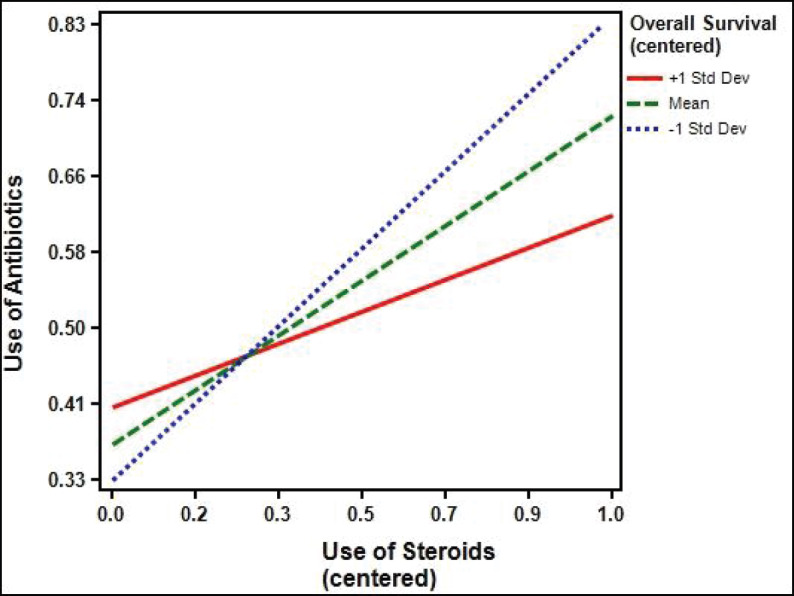
Interaction between use of antibiotics and use of steroids on their effect on overall survival (*p* = 0.016).

The median follow up duration was 9.5 months (95% CI: 6.8–12.2). Median PFS in patients who received antibiotics was 1.7 months (95% CI: 1.1–2.3) as against 3.6 months (95% CI: 2.3–4.8) for patients who did not receive antibiotics (*p* = 0.912). Median OS in the patients who received antibiotics was 3.9 months (95% CI: 1.8–11.4) as compared to 9.2 months (95% CI: 4.2–12.3) who did not receive antibiotics *p* = 0.053 (HR = 1.023; 95%CI: 1.00–1.04). The percentage of patients surviving at 12 months who didn’t receive antibiotics was 39.4% (±6.7) versus 36.2% (±6.4) for those who received antibiotics. Among the patients who received antibiotics, median OS for patients who received ≤10 days of antibiotics was 8.8 months (95% CI: 4.2–11.2) while for patients receiving >10 days of antibiotics, it was 2.8 months (95% CI: 1.2–4.4), *p* = 0.025 (HR 2.0, 95% CI: 1.1–3.7, Figure 2). One-year survival for the corresponding groups was 47.3% (±9.2) and 22.2 % (±7.9), respectively. Median OS for patients with RCC as primary was 5.3 months (95% CI: 1.8–8.8, *p* = 0.225). For urothelial carcinoma as primary, median OS was 5 months (95% CI: 0–12.6, *p* = 0.899). On univariate analysis, performance status, use of steroids and duration of antibiotics use came as significant factors and all the three factors were found to be significant in the multivariate analysis also (Table 1). In addition to antibiotics, the patients who also received concomitant steroids (10 mg or more of prednisolone equivalent) had median OS of 2.8 months (95% CI: 1.5–4.1) versus 5.4 months (95% CI: 3.2–7.4) for those who did not receive steroids (HR 1.9, 95% CI 1.0–3.6, *p* = 0.034, Figure 3). Patients with PS 0–1 had median OS of 8.8 months (95% CI: 4.4–13.7) while it was 1.9 months (95% CI: 1.3–5.2) for patients with PS 2–4 (HR 2.2, 95% CI: 1.2–4.2, *p* = 0.012). There was no significant difference in survival based on the class of antibiotics used, gender, age, site of primary, line of therapy and presence of brain metastasis. There was a statistically significant interaction between the effects of use of steroids and use of antibiotics on the overall survival, *F* (1,151) = 5.9, *p* =0.016 (Figure 4).

Thirty-three (21.2% of total) patients received antibiotics during the window of 2 weeks before the start of ICI to 2 months of starting ICI. Median OS in the patients who received antibiotics in this window was 2.8 months (95% CI: 1.2–4.5) as compared to 9.2 months (95% CI: 5.2–13.1) who did not receive antibiotics *p* = 0.008 (HR = 1.8; 95% CI: 1.2–3.0). The percentage of patients surviving at 12 months who didn’t receive antibiotics in this particular time-window was 41.8% (±5.7) versus 22.5% (±7.4) for those who received antibiotics.

## Discussion

In a systematic review by Elkrief *et al* [1], 11 out of the 12 retrospective studies suggested a negative impact of antibiotics on clinical outcome of patients with NSCLC, RCC or melanoma who were treated with ICIs. This study also suggested that treatment with broad-spectrum antibiotics during the month before the commencement of ICI appears particularly deleterious. Our study also showed a trend towards poorer survival in patients who received antibiotics during ICI and subgroups of patients who received antibiotics for more than 10 days had significantly poorer outcomes as compared to antibiotics for 10 days or less. Also, clinical trials are currently underway to evaluate the utility of targeted interventions designed to rapidly revert antibiotic-induced dysbiosis by employing FMT, prebiotics, and probiotics [1].

In our study, we included patients who received antibiotics up to 2 weeks before the start of ICI. Previous studies have suggested a time-dependent and partial repopulation of the gut microbiota after discontinuation of antibiotics [7]. In the only negative study for antibiotics association with clinical outcomes in patients treated with ICI, 74 NSCLC patients treated with nivolumab were included, the antibiotic-treated arm included patients receiving antibiotics up to 3 months before ICI treatment which is a relatively long period as compared to other studies [8]. Also, it only included 20.3% of all patients, which is very low as compared with other studies that included antibiotics treated patients until 2 months or less [8]. In our study, 44% of overall patients received antibiotics which are in harmony with various other studies [1]. Based on results of previous studies, the antibiotic window of 2 weeks before the start of ICI was considered optimal and thus, this cut off was used for selecting the antibiotic window. Also, an additional analysis was performed to evaluate the use of antibiotics during first 2 months versus other patients receiving ICI. This cut off was chosen as first response scan was done in most of the patients after four doses (2 months) of the ICI therapy. When this time-window was used, the difference in overall survival reached statistical significance. This might be explained by the phenomena called ‘immortal patient bias’ since patients who responded to ICI have more chances of receiving antibiotics during their entire treatment.

In our study, we found that a longer duration of antibiotics use was associated with poorer clinical outcomes and this was independent of performance status. Another important finding in our study was statistically significant better OS in patients who did not receive steroids versus those who did. To our knowledge, no previous study has reported results on the use of steroids in patients who received antibiotics while receiving ICI. Thus, our study adds important information on the interaction of the use of both antibiotics and steroids in patients receiving ICI. This interaction was found to be statistically significant affecting the overall survival. Another important strength of this study being the use of single ICI in fairly large number of patients as against other studies which report the use of multiple ICIs in smaller cohorts of patients. Besides, this study demonstrated statistically significant interaction between the effects of use of steroids and use of antibiotics on the overall survival.

There are some important drawbacks of the study; apart from the retrospective nature of the study, this study included principally patients of NSCLC and head neck cancers. This may preclude the broad application of results to every solid tumour treated with ICI. Also, breaking down the data into various sub-groups might have led to non-significant results making interpretation difficult. Despite these limitations, this study adds important real-world data on the use of antibiotics concomitant with ICI.

## Conclusions

This study shows that the judicious use of antibiotics is required in patients on ICI or scheduled to be started on ICI. However, more data is required for recommending postponement of initiation of ICI which would allow a spontaneous recovery from antibiotic mediated dysbiosis.

## Conflicts of interest

The authors have no conflicts of interest to declare.

## Source of funding

The authors or institution did not receive any funding for this research.

## Authors' contributions

Conception and design: Vanita Noronha, Kumar Prabhash. Provision of patients: Vanita Noronha, Amit Joshi, Vijay Maruti Patil, Amit Joshi, Nandini Menon, Kumar Prabhash. Collection and assembly of data: Akhil Kapoor, Abhishek Mahajan, Amit Janu. Data analysis and interpretation: Akhil Kapoor, Vanita Noronha, Kumar Prabhash. Manuscript writing: All authors. Final approval of manuscript: All authors. Accountable for all aspects of the work: All authors.

## Figures and Tables

**Table 1.** Univariate and multivariate analysis of factors among patients who received antibiotics.

Factor	Sub-factors	*n* (%)	Median OS (95% CI)	*p* value	Mutivariate analysis HR (95% CI)	*p* value
Gender	Female	12 (17.1)	1.9 (1.7–2.0)	0.076		
	Male	58 (82.9)	4.4 (2.4–6.4)			
ECOG PS	0–1	37 (52.8)	8.8 (4.4–13.7)	**0.012**	1	**0.007**
	2–4	33 (47.1)	1.9 (1.3–5.2)		2.4 (1.3–4.7)	
Line of therapy	1–2	36 (51.4)	3.7 (1.7–5.8)	0.288		
	3 or more	34 (48.6)	5.0 (1.8–7.4)			
Age	<60 years	45 (64.3)	3.7 (1.1–6.4)	0.707		
	≥60 years	25 (35.7)	5.0 (2.2–7.8)			
Brain metastasis	No	64 (91.4)	4.4 (2.5–6.3)	0.054		
	Yes	06 (8.6)	0.7 (0–2.4)			
Steroids Use	No	43 (61.4)	5.4 (3.2–7.4)	**0.034**	1	**0.035**
	Yes	27 (38.6)	2.8 (1.5–4.1)		1.9 (1.0–3.6)	
Duration of Antibiotics use	10 days or less	41 (58.6)	8.8 (4.6–10.8)	**0.025**	1	**0.026**
	>10 days	29 (41.4)	2.8 (1.2–4.4)		2.0 (1.0-3.8)	
Site of primary	Lung	34 (63.1)	2.9 (0.3–5.5)	0.769		
	Head & neck	20 (15.8)	3.9 (0.8–6.9)			
	Others	16 (21.0)	5.3 (1.1–9.6)			
Class of Antibiotics	Penicillin (P)	31 (65.7)	5.4 (4.1–6.8)	0.236		
	Fluoroquinolone (FQ)	5 (11.4)	NR			
	Carbapenem	6 (8.6)	1.9 (0–4.1)			
	P + FQ	18 (25.7)	1.5 (0–4.9)			
	Others	10 (14.2)	2.7 (1.6–3.8)			

OS: Overall survival; HR: Hazard ratio; CI: Confidence interval; ECOG PS: Eastern cooperative oncology group Performance status; NR: Not reached.
